# Effects of six different microbial strains on polyphenol profiles, antioxidant activity, and bioaccessibility of blueberry pomace with solid-state fermentation

**DOI:** 10.3389/fnut.2023.1282438

**Published:** 2023-10-12

**Authors:** Zhu-Xi Tian, Yong-Fu Li, Ming-Xiu Long, Qian Liang, Xi Chen, Dao-Mei Huang, Yao-Qi Ran

**Affiliations:** Guizhou Institute of Integrated Agricultural Development, Guizhou Academy of Agricultural Sciences, Guiyang, China

**Keywords:** blueberry pomace, solid-state fermentation, simulated gastrointestinal digestion, antioxidant activity, polyphenol profiles

## Abstract

To explore the effect of different microbial strains on blueberry pomace with solid-state fermentation (SSF), three fungi strains and three lactic acid bacteria (LAB) strains were utilized to investigate with respect to polyphenol profiles, antioxidant capacities, and bioaccessibility. Different strains exhibited different capacities for metabolizing polyphenolic compounds in blueberry pomace. The contents of 10 phenolic acids and 6 flavonoids (except (+)-catechin) were increased in blueberry pomace fermented by *Lactobacillus acidophilus* (LA). A similar tendency was observed in blueberry pomace fermented by *Aspergillus niger* (AN) and *Lactobacillus plantarum* (LP), where the concentration of 8 phenolic acids and 5 flavonoids was enhanced, with the following exceptions: (+)-catechin, ferulic acid, vanillic acid, and quercitrin. Chlorogenic acid and quercetin were the maximum phenolic acids and flavonoids in blueberry pomace with SSF, upgraded at 22.96 and 20.16%, respectively. Contrary to the growth of phenolic acids and flavonoid compounds, all individual anthocyanins showed a decreased trend. Only in the blueberry pomace fermented by AN, all anthocyanidins exhibit a rising trend. After SSF, 2,2′-azino-bis (3-ethylbenzothiazoline-6-sulfonic acid) (ABTS), 2,2-diphenylpicrylhydrazyl (DPPH), and ferric reducing antioxidant power (FRAP) radical scavenging abilities were increased by up to 33.56, 59.89, and 87.82%, respectively. Moreover, the simulated gastrointestinal digestion system revealed that SSF improved the bioaccessibility of polyphenolic compounds. Compared with other strains, LA, LP, and AN showed better excellent capacities for metabolizing polyphenolic compounds, which led to a greater increase in antioxidant activity and bioaccessibility in fermented blueberry pomace.

## Introduction

A growing interest has been focused on polyphenol compounds for their capacity for biological activities. Blueberry is generally recognized as an ideal source of polyphenol compounds and contains large quantities of phenolic acids, flavonols, anthocyanins, and other polyphenol compounds (1, 2). However, blueberry is susceptible to spoilage with a short shelf life, due to moisture loss, microbial rot, and bruising damage (3). Therefore, a large amount of blueberry fruits is widely processed into juices, wines, jams, and other products to improve the economic value of blueberry. During the production process, mountains of blueberry pomace are manufactured as an inevitable by-product. There is a growing interest in utilizing fruit and food by-products as ingredients of bioactive substances for healthy products (4). Studies have reported that a considerable part of anthocyanins and polyphenols is still available in the blueberry pomace; only a small amount is presented in blueberry products, which gives blueberry pomace a wide range of bioactivities and health effects (5, 6). Therefore, the application of blueberry pomace as a nutritional food material is highly promising (3, 6). Several methods have emerged to produce polyphenol-rich products from pomace, such as acid hydrolysis (7), enzymatic hydrolysis (8), microbial fermentation (9), and ultrasound-assisted extraction (10). Among them, microbial fermentation is a comprehensive, low toxicity, cost-effectiveness, and environmental-friendly approach to enrich polyphenol profiles and improve antioxidant activity, which has obtained considerable attention lately (4, 9).

Microorganisms (e.g., fungi and lactic acid bacteria) have been proven to secrete carbohydrate-degrading enzymes that effectively break cell wall, hydrolyze glycosidic bond, and release bound polyphenols (11–13). Several studies have reported that the fermentation of diverse types of pomace (apple pomace, bayberry pomace, and grape pomace) by various microorganisms promotes higher antioxidant activity because of the increase in total polyphenol content (14–16). Yan et al. (3) demonstrated that in blueberry pomace by SSF with *L. rhamnosus* GG and *L. plantarum*-1, the phenolic content increased remarkably along with a subsequent improvement in antioxidant capacity. Similarly, fermentation of *Lactobacillus casei* increased polyphenol content in blueberry pomace and enriched the blueberry pomace with 13 more phenolic compounds than the unfermented sample, which was positively associated with antioxidant activity and gut-improving function.

The fermented blueberry pomace could be a possible ingredient for functional foods (9). Published research studies on blueberry pomace have mainly focused on lactic acid bacteria fermentation and submerged fermentation, but little information is available on the fungal and solid-state fermentation alteration of polyphenol compounds and antioxidant capacities of blueberry pomace (3, 9, 10). Compared with submerged fermentation, solid-state fermentation (SSF) has shown a higher concentration of the product, less wastewater generation, and lower catabolic repression (17, 18). Therefore, the current study was conducted to explore the influence of the selected microbial strains on polyphenol profiles, antioxidant capacities, and bioaccessibility of blueberry pomace with solid-state fermentation. Furthermore, the relationships between individual phenolic compounds and their antioxidant capacities were assessed.

## Materials and methods

### Materials

Fresh blueberries were bought from local farms in Majiang, Guizhou province, China, and transported to our laboratory immediately. The blueberries were washed and pressed to remove the juice. The pomace (consisting of peel and cores) was separated from the blueberry juice and stored at −20°C for further study.

### Microorganisms and culture conditions

The following commercial microbial strains were purchased from China Center of Industrial Culture Collection: *Aspergillus niger* CICC 40493 (AN), *Aspergillus oryzae* CICC 2400 (AO), *Monascus anka* CICC 5013 (MA), *Lactobacillus plantarum* CICC 20265 (LP), *Lactobacillus acidophilus* CICC 20244 (LA), and *Lactobacillus casei* CICC 23184 (LC). The three fungal strains and the three LAB strains were maintained on potato dextrose agar (PDA) slants and de Man–Rogosa–Sharpe agar (MRS) slants at 4°C, respectively. The fungal spores were counted using a blood cell counting chamber. The LAB strains were cultivated for 16 h at 37°C before use.

### SSF of blueberry pomace

In total, 20 g blueberry pomace and 14 mL distilled water were inoculated with LAB strain (10^7^ CFU/g) at 37°C for 3 days and fungal spore suspension (10^7^ spore/g) at 30°C for 5 days, respectively. After SSF, all samples were lyophilized and stored at −80°C until use.

### Extraction of polyphenolic compounds

Polyphenolic compounds from dried pomace powder (0.5 g) were extracted three times with 20 mL of extraction mixture (6M hydrochloric acid:methanol:water in the ratio 1:70:29 ) at 40°C for 30 min in an ultrasonic bath (19). The mixture was centrifuged at 12,000 rpm for 10 min. The supernatants were evaporated until dry and stored in methanol at 4°C until analysis.

### Determination of polyphenol profiles by HPLC-DAD analysis

The measurement of polyphenol profiles was conducted on an Agilent 1260 HPLC with a DAD detector, coupled to an MS detector single quadrupole Agilent 6110. Chromatographic analysis was performed on a 250 × 4.6 mm, 3 μm particle size, C18 column (YMC-Pack ODS-AQ, Japan). Mobile phase A was 5% (v/v) aqueous formic acid and mobile phase B was 100% acetonitrile. The gradient elution program was as follows: 0 min, 5% B; 0–5 min, 5%; 5–30 min, 15% B; 30–50 min, 25% B; 50–60 min, 80% B; 60–75 min, 80% B; 75–85 min, 5% B. The column was then washed and reconditioned. The injection volume, flow rate, and running temperature were maintained at 10 μL, 1 mL/min, and 25°C, respectively. The compounds were monitored at 280, 320, 360, and 520 nm.

### Phytochemical concentration assay

#### Total phenolic concentration

The TPC of the extracts was measured by the Folin–Ciocalteu method (20) with minor modifications. In total, 125 μL of extract solution was mixed with 0.5 mL of water and 125 μL of freshly prepared Folin–Ciocalteu reagent for 6 min in darkness. Then, 1.25 mL of 7.5% Na_2_CO_3_ (w/v) was subsequently added. The mixture was reacted at 30°C for 90 min. Then, its absorbance was determined at 760 nm (Thermo Genesys 10S spectrophotometer, Thermo Fisher Scientific, USA). The results were expressed as milligrams of gallic acid equivalents/gram DW of pomace (mg GAE/g).

#### Total flavonoid concentration

The TFC of the extracts was evaluated according to the AlCl_3_ colorimetric method with slight modifications (15). In brief, 300 μL of extract solution was added to 1.5 mL deionized water and 90 μL of 5% NaNO_2_ solution for 6 min. After that, 180 μL of AlCl_3_ solution (100 g/L) was combined with the mixture and stood for 5 min. Subsequently, 600 μL of NaOH (1.0 M) and 330 μL of distilled water were added. The absorbance was measured at 510 nm after 10 min. The results were expressed as rutin equivalents/gram DW of pomace (mg RE/g).

#### Total anthocyanin concentration

The TAC of the extracts was determined using the differential pH method with slight modifications (15, 21). The extracts were mixed with buffer pH 1.0 (0.025 M KCl) and buffer pH 4.5 (0.4 M NaAc), respectively. After reacting for 15 min in darkness, the absorbance was read at 510 and 700 nm. The content of anthocyanin was expressed as milligrams of cyanidin-3-glucoside equivalent per gram of pomace (mg C3G/g DW).

### Analysis of antioxidant activities

#### Determination of DPPH radical scavenging activity

A slight modification of the method, previously reported by Macedo et al. (20), was used to assess the DPPH radical scavenging activity. The reaction system contained 3.9 mL DPPH methanol solution (24 mg/L) and 0.1 mL polyphenolic extract solution. After a reaction of 30 min, the absorbance was determined at 517 nm. The results were expressed as μmol Trolox/g using Trolox as a standard.

#### Determination of FRAP radical scavenging activity

The ferric reducing antioxidant power (FRAP) assay was determined by the method by Yan et al. (3). Fresh FRAP working reagent was prepared by mixing 0.3 M acetate buffer (30 mL), 0.01 mM TPTZ (2,4,6-tripyridyl-s-triazine) solution (3.0 mL), and 0.02 mM FeCl_3_ solution (3.0 mL) and preheated in a water bath at 37°C prior to use. Then, 0.1 mL of polyphenolic extract solution was added to 3 mL of the FRAP working reagent at 37°C for 10 min. The absorbance was detected at 593 nm. The results were expressed as μmol Trolox/g using Trolox as a standard.

#### Determination of ABTS radical scavenging activity

ABTS radical scavenging activity was determined according to a method previously reported by Macedo et al. (20), with slight modifications. An equal volume of ABTS solution (7.4 mM) and K_2_S_2_O_8_ (2.6 mM) were mixed and reacted for 16 h. Then, this mixture was added with methanol to adjust the absorbance of 0.70 ± 0.02 at 734 nm. Afterward, 150 μL of polyphenolic extract solution was added to 0.85 mL of the mixture. The absorbance was determined at 734 nm after 6 min. The results were expressed as μmol Trolox/g using Trolox as a standard.

### *In vitro* simulated gastrointestinal digestion

The *in vitro* simulated gastrointestinal digestion was used to evaluate the bioaccessibility of polyphenolic compounds according to the modified methods by Brodkorb et al. (22) and Gao et al. (23). All the simulated salivary fluids, simulated gastric fluids (SGF), and simulated intestinal fluids (SIF) were made up of KH_2_PO_4_ (0.5 M), KCl (0.5 M), NaCl (2.0 M), CaCl_2_(H_2_O)2 (0.3 M), MgCl_2_(H_2_O)_6_ (0.15 M), NaHCO_3_ (1.0 M), (NH_4_)_2_CO_3_ (0.5 M), and HCL (6.0 M).

#### Oral phase

Five grams of blueberry pomace was mixed with 4 mL simulated salivary fluids, 0.025 mL CaCl_2_(H_2_O)_2_ (0.3 M), and 0.925 mL water. These mixtures were incubated at 37°C for 2 min.

#### Gastric phase

The oral bolus was mixed with 8 mL SGF, 0.005 mL CaCl_2_(H_2_O)_2_ (0.3 M), 0.667 mL pepsin (20 mg/mL), and 0.48 mL gastric lipase (100 mg/mL). These mixtures were altered to pH 3.0 by adding 5 M NaOH solution and were incubated at 37°C for 120 min.

#### Intestinal phase

The gastric system was combined with 8 mL SIF and 0.04 mL CaCl_2_(H_2_O)_2_ (0.3 M). Then, 5 M NaOH was utilized to reach pH 7.0 before the addition of 5 mL pancreatin (133.3 mg/mL) and 3 mL bile salt (200 mg/mL). A dialysis bag, including 5.5 mL 1.0 M NaHCO_3_ and 5.5 mL normal saline, was immersed in the simulated small intestine. These mixtures were further incubated at 37°C. The solutions from the interior (absorbent) and exterior (gastrointestinal) parts of the dialysis bag were then collected.

Bioaccessibility was calculated using the following equation:


$$\begin{array}{l} PAV\;\left(\%\right)\;=\;A_{IN}\;÷A_{non-digested} \end{array}$$

where A_non−digested_ is the content of polyphenolic compounds in non-digested samples (mg/100 g), and A_IN_ is the content of polyphenolic compounds in the dialysis bag (mg/100 g).

### Statistical analysis

All the measurements were conducted three times. The data were performed as mean ± standard deviation (SD). Correlation analysis was measured by Pearson's correlation test in Origin 2022. Significant differences were calculated by the Duncan test in IBM SPSS Statistics 22.0, and *p* < 0.05 was considered statistically significant. Principal component analysis (PCA) was determined in Origin 2022.

## Results

### Effect of SSF on individual anthocyanin and anthocyanidin composition of blueberry pomace

Ten anthocyanins and five anthocyanidins were identified and quantified in the blueberry pomace extracts (Table 1). As shown in Table 1, delphinidin-3-O-galactoside, petunidin-3-O-glucoside, and malvidin-3-O-glucoside were the major anthocyanin compounds, and malvidin was the major anthocyanidin compound in all blueberry pomace samples. The contents of all anthocyanins were declined by SSF with different microbial strains. Additionally, the total content of anthocyanins in the AN group was the lowest, decreased by 42.99% compared with the control group. The maximum total content of anthocyanins was observed in the MA group, decreased by 26.30% compared with the control group. In terms of anthocyanidin, delphinidin and peonidin were not detected in the control group but were found in the LP, LA, and AN groups. The anthocyanidin profile presented complex changes. Only in the AN group, all anthocyanidin substances present a growth trend. The highest concentration of cyanidin, petunidin, malvidin, delphinidin, and peonidin was also observed in the AN group at 17.29 ± 0.48, 13.63 ± 0.13, 33.63 ± 1.10, 18.25 ± 1.03, and 14.44 ± 0.04 mg/100 g, respectively. The total content of anthocyanidins declined by 51.18 and 50.18% in the LC and MA groups, respectively, compared with the control group. In the other fermented blueberry pomace groups, the total content of anthocyanidins was increased. Especially, the maximum total content of anthocyanidins was observed in the AN group, which increased by 58.47% compared with the control group. These results demonstrated that *Aspergillus niger* exerted a stronger capability than other strains for transforming anthocyanins into anthocyanidins.

**Table 1:** Profiles of anthocyanin and anthocyanidin in different blueberry pomace samples (mg/100 g).

**Compounds**	**Control**	**LA**	**LP**	**LC**	**AO**	**AN**	**MA**
Delphinidin-3-O-galactoside	71.34 ± 6.78^a^	31.79 ± 1.55^cd^	26.59 ± 3.50^d^	29.25 ± 1.37^cd^	36.59 ± 3.87^bc^	24.70 ± 3.89^d^	41.72 ± 5.32^b^
Delphinidin-3-O-glucoside	14.24 ± 0.30^a^	11.95 ± 0.36^c^	11.21 ± 0.30^d^	12.74 ± 0.02^b^	12.40 ± 0.31^bc^	11.97 ± 0.27^c^	12.43 ± 0.09^bc^
Cyanidin-3-O-galactoside	12.81 ± 0.60^a^	7.21 ± 0.81^de^	6.90 ± 0.73^e^	10.47 ± 0.07^b^	9.52 ± 0.98^bc^	8.37 ± 0.45^cd^	9.46 ± 0.65^bc^
Cyanidin-3-O-glucoside	21.04 ± 0.57^a^	17.40 ± 0.95^b^	14.78 ± 0.96^d^	18.62 ± 0.11^b^	17.81 ± 0.73^b^	16.13 ± 0.48^c^	17.73 ± 0.39^b^
Petunidin-3-O-galactoside	16.33 ± 0.05^a^	16.33 ± 0.13^a^	16.13 ± 0.23^a^	16.08 ± 0.01^a^	16.30 ± 0.31^a^	nd	16.10 ± 0.07^a^
Petunidin-3-O–glucoside	39.97 ± 2.31^a^	24.88 ± 2.26^cd^	23.69 ± 2.97^cd^	29.40 ± 0.62^b^	26.69 ± 2.94^bc^	21.31 ± 1.49^d^	25.51 ± 1.25^bcd^
Peonidin-3-O-glucoside	21.90 ± 0.51^a^	17.56 ± 0.71^de^	16.94 ± 0.81^e^	19.89 ± 0.04^b^	18.94 ± 0.81^bc^	18.22 ± 0.43^cd^	19.12 ± 0.47^bc^
Malvidin-3-O-galactosid	27.19 ± 1.12^a^	20.78 ± 2.67^bc^	20.80 ± 0.97^bc^	23.19 ± 0.51^b^	21.54 ± 2.01^bc^	18.56 ± 0.82^c^	20.59 ± 2.49^bc^
Malvidin-3-O-glucoside	31.52 ± 1.23^a^	23.19 ± 1.76^bc^	23.68 ± 1.75^bc^	25.53 ± 0.02^b^	23.64 ± 1.77^bc^	21.03 ± 1.17^c^	22.90 ± 0.83^bc^
Malvidin-3-O-arabinoside	17.19 ± 0.17^a^	17.02 ± 0.34^a^	16.20 ± 0.53^b^	16.05 ± 0.03^b^	16.11 ± 0.63^b^	15.68 ± 0.12^b^	16.01 ± 0.20^b^
Delphinidin	nd	11.81 ± 0.45^d^	13.50 ± 0.10^c^	nd	10.95 ± 0.65^d^	18.25 ± 1.03^a^	15.21 ± 0.27^b^
Cyanidin	15.32 ± 0.05^b^	13.32 ± 0.58^c^	14.81 ± 0.37^b^	16.47 ± 0.75^a^	14.29 ± 0.60^bc^	17.29 ± 0.48^a^	15.35 ± 0.77^b^
Petunidin	13.25 ± 0.10^a^	10.31 ± 0.67^c^	11.50 ± 0.14^b^	13.48 ± 0.03^a^	10.38 ± 0.91^c^	13.63 ± 0.13^a^	nd
Peonidin	nd	11.42 ± 0.37^c^	12.57 ± 0.04^b^	nd	nd	14.44 ± 0.04^a^	nd
Malvidin	32.78 ± 0.33^a^	30.66 ± 0.58^b^	32.96 ± 0.06^a^	nd	28.73 ± 1.11^c^	33.63 ± 1.10^a^	nd
Total anthocyanins	273.53 ± 13.44^a^	188.11 ± 5.24^bc^	176.92 ± 3.20^c^	201.21 ± 1.78^b^	199.54 ± 6.47^b^	155.95 ± 9.04^d^	201.58 ± 11.12^b^
Total anthocyanidins	61.36 ± 0.93^e^	77.52 ± 1.66^c^	85.34 ± 0.64^b^	29.96 ± 0.72^f^	64.34 ± 0.81^d^	97.24 ± 2.54^a^	30.57 ± 1.01^f^

Different letters in a row indicate significant differences (*p* < 0.05). nd, not detected. Values are means of triplicate samples and expressed as mean ± SD.

LA, *Lactobacillus acidophilus*; LP, *Lactobacillus plantarum*; LC, *Lactobacillus casei*; AO, *Aspergillus oryzae*; AN, *Aspergillus niger*; MA, *Monascus anka*.

### Effect of SSF on individual phenolic acid and flavonoid composition of blueberry pomace

The amount of 10 phenolic acids and 7 flavonoids in fermented blueberry pomace was evaluated (Table 2). Chlorogenic acid and quercetin were the primary phenolic acids and flavonoids in blueberry pomace, with the content ranging from 17.76 ± 1.60 mg/100 g (AO) to 26.46 ± 2.09 mg/100 g (AN) and from 62.53 ± 5.81 mg/100 g (AO) to 86.47 ± 3.07 mg/100 g (LA), increased by up to 22.96 and 20.16% compared with the control group, respectively. After SSF, the contents of gallic acid, protocatechuic acid, *p*-hydroxybenzoic acid, gentisic acid, caffeic acid, *p*-coumaric acid, rutin, and epicatechin of six fermented blueberry pomaces were increased. Among them, protocatechuic acid and epicatechin were the phenolic acids and flavonoids with the highest growth rates, increased by 3.51-fold in the AN group and 2.13-fold in the LP group, respectively. Meanwhile, the content of (+)-catechin in all fermented blueberry pomace samples was reduced by 0.09–0.36%. *p*-Hydroxybenzoic acid was not detected in the control group but was found in all fermented blueberry pomaces and achieved the maximum content of 3.34 ± 0.52 mg/100 g in the LP group. Additionally, the increasing trends of all phenolic acids and flavonoids were observed in the LA group after SSF, except for (+)-catechin. A similar tendency was also found in the AN and LP groups, where the contents of all phenolic acids and flavonoids were increased, except for (+)-catechin, ferulic acid, vanillic acid, and quercitrin. After SSF, the total contents of phenolic acids and flavonoids were enhanced in all fermented blueberry pomaces. Furthermore, the total contents of phenolic acids and flavonoids were significantly increased in the LA, LP, and AN groups, whereas there was no significant difference among them. These results perhaps illustrated that *Lactobacillus acidophilus, Lactobacillus plantarum*, and *Aspergillus niger* exerted greater capabilities for enhancing the content of phenolic acids and flavonoids than the other strains.

**Table 2:** Profiles of phenolic acid and flavonoid in different blueberry pomace samples (mg/100 g).

**Compounds**	**Control**	**LA**	**LP**	**LC**	**AO**	**AN**	**MA**
Gallic acid	2.23 ± 0.37^d^	5.47 ± 0.40^a^	4.78 ± 0.58^ab^	3.57 ± 0.42^c^	5.07 ± 0.21^a^	3.98 ± 0.38^c^	4.06 ± 0.30^bc^
Protocatechuic acid	1.82 ± 0.22^c^	7.22 ± 0.30^ab^	7.86 ± 0.46^a^	6.51 ± 0.23^b^	6.69 ± 0.50^b^	8.21 ± 0.99^a^	6.68 ± 0.36^b^
*p*-Hydroxybenzoic acid	nd	3.13 ± 0.54^a^	3.34 ± 0.52^a^	1.43 ± 0.19^b^	0.87 ± 0.01^b^	2.65 ± 0.47^a^	1.70 ± 0.85^b^
Gentisic acid	7.82 ± 0.60^d^	10.17 ± 0.25^a^	9.65 ± 0.36^ab^	8.89 ± 0.40^bc^	8.38 ± 0.50^cd^	9.02 ± 0.50^bc^	7.92 ± 0.62^d^
Chlorogenic acid	21.52 ± 0.42^bc^	26.38 ± 2.19^a^	22.74 ± 0.86^b^	18.96 ± 1.00^cd^	17.76 ± 1.60^d^	26.46 ± 2.09^a^	18.51 ± 1.03^d^
Vanillic acid	2.56 ± 0.08^bc^	3.52 ± 0.36^a^	2.01 ± 0.38^c^	3.39 ± 0.18^a^	2.18 ± 0.20^c^	3.20 ± 0.62^ab^	2.61 ± 0.29^bc^
Caffeic acid	0.47 ± 0.12^c^	0.75 ± 0.13^ab^	0.73 ± 0.13^ab^	0.61 ± 0.04^bc^	0.92 ± 0.15^a^	0.80 ± 0.19^ab^	0.88 ± 0.08^a^
Syringic acid	0.96 ± 0.25^d^	2.92 ± 0.31^c^	4.35 ± 0.86^b^	2.57 ± 0.99^c^	2.77 ± 0.32^c^	5.95 ± 0.88^a^	0.92 ± 0.20^d^
*p*-Coumaric acid	2.18 ± 0.17^b^	3.02 ± 0.01^a^	2.97 ± 0.03^a^	3.02 ± 0.03^a^	3.06 ± 0.18^a^	2.97 ± 0.02^a^	2.95 ± 0.03^a^
Ferulic acid	0.09 ± 0.02^b^	0.13 ± 0.02^a^	0.13 ± 0.02^a^	0.08 ± 0.02^b^	0.01 ± 0.01^c^	0.04 ± 0.01^c^	nd
Rutin	1.58 ± 0.46^b^	2.47 ± 0.33^a^	1.89 ± 0.20^ab^	2.33 ± 0.20^a^	1.85 ± 0.27^ab^	2.49 ± 0.42^a^	1.65 ± 0.35^b^
Hyperoside	2.54 ± 0.05^ab^	2.91 ± 0.21^a^	2.96 ± 0.43^a^	2.55 ± 0.13^ab^	2.69 ± 0.22^ab^	2.65 ± 0.16^ab^	2.27 ± 0.08^b^
Luteoloside	3.12 ± 0.01^b^	5.50 ± 0.35^a^	4.85 ± 1.25^a^	2.89 ± 0.19^b^	3.14 ± 0.12^b^	4.85 ± 1.00^a^	2.56 ± 0.19^b^
Quercitrin	13.68 ± 0.21^ab^	15.02 ± 0.52^a^	13.51 ± 1.28^ab^	13.24 ± 0.94^b^	12.81 ± 0.66^bc^	13.88 ± 0.91^ab^	11.59 ± 0.57^c^
Quercetin	71.96 ± 3.27^cd^	86.47 ± 3.07^a^	80.59 ± 2.28^ab^	67.88 ± 4.76^de^	62.53 ± 5.81^e^	78.68 ± 2.51^bc^	75.35 ± 3.83^bc^
Epicatechin	11.70 ± 1.64^c^	32.66 ± 5.23^ab^	36.63 ± 6.26^a^	26.10 ± 1.55^b^	28.71 ± 8.10^ab^	29.85 ± 1.50^ab^	17.14 ± 2.00^c^
(+)-Catechin hydrate	17.37 ± 0.62^a^	13.52 ± 1.81^bc^	13.98 ± 0.75^bc^	13.12 ± 2.01^bc^	12.60 ± 2.14^bc^	15.75 ± 1.62^ab^	11.11 ± 1.52^c^
Total content	161.60 ± 5.63^b^	221.27 ± 11.99^a^	213.33 ± 9.55^a^	177.43 ± 3.24^b^	172.05 ± 13.38^b^	210.81 ± 5.50^a^	167.89 ± 3.47^b^

Different letters in a row indicate significant differences (*p* < 0.05). nd, not detected. Values are means of triplicate samples and expressed as mean ± SD.

LA, *Lactobacillus acidophilus*; LP, *Lactobacillus plantarum*; LC, *Lactobacillus casei*; AO, *Aspergillus oryzae*; AN, *Aspergillus niger*; MA, *Monascus anka*.

### Effect of SSF on TPC, TFC, and TAC in blueberry pomace

As shown in Table 3, TPC and TFC in fermented blueberry pomace groups were significantly higher than those in the control samples. A range of 167.67 ± 7.95 mg GAE/g DW (CON) to 209.18 ± 7.60 mg GAE/g DW (AN) was obtained for the TPC. The highest TPC was observed in AN and increased by 24.75% after fermentation. The TFC showed a similar trend. The results of TFC ranged from 43.51 ± 2.39 mg RT/g DW (CON) to 93.71 ± 2.07 mg RT/g DW (LA). TFC in all fermented blueberry pomace samples was dramatically higher than that in the CON. The TFC in LA was the highest, increased by 1.15-fold after fermentation. In contrast, the TAC was decreased from 8.94 ± 0.56 mg C3G/g DW (CON) to 4.99 ± 0.73 mg C3G/g DW (MA). Similar to the above results, SSF led to an increase in TPC and TFC and a decline in TAC.

**Table 3:** The content of total phenolic, total flavonoid, and total anthocyanin in different blueberry pomace samples.

	**TPC (mg GAE/g DW)**	**TFC (mg RT/g DW)**	**TAC (mg C3G/g DW)**
Control	167.67 ± 7.95^d^	43.51 ± 2.39^d^	8.94 ± 0.56^a^
LA	194.39 ± 1.18^bc^	93.71 ± 2.07^a^	7.81 ± 0.53^b^
LP	190.71 ± 5.43^bc^	80.71 ± 6.19^b^	7.58 ± 0.37^bc^
LC	188.73 ± 2.15^c^	84.10 ± 7.61^b^	7.70 ± 0.49^b^
AO	199.13 ± 2.90^b^	83.55 ± 1.58^b^	6.65 ± 0.56^c^
AN	209.18 ± 7.60^a^	85.75 ± 3.17^b^	6.90 ± 0.28^bc^
MA	171.35 ± 4.32^d^	62.84 ± 2.92^c^	4.99 ± 0.73^d^

Different letters in a column indicate significant differences (*p* < 0.05). Values are means of triplicate samples and expressed as mean ± SD.

LA, *Lactobacillus acidophilus*; LP, *Lactobacillus plantarum*; LC, *Lactobacillus casei*; AO, *Aspergillus oryzae*; AN, *Aspergillus niger*; MA, *Monascus anka*; GAE, gallic acid equivalent; RT, rutin; C3G, cyanidin-3 glucoside; TPC, total phenolic content; TFC, total flavonoid content; TAC, total anthocyanin content.

### Effect of SSF on the antioxidant activity in blueberry pomace

**Figure 1:**
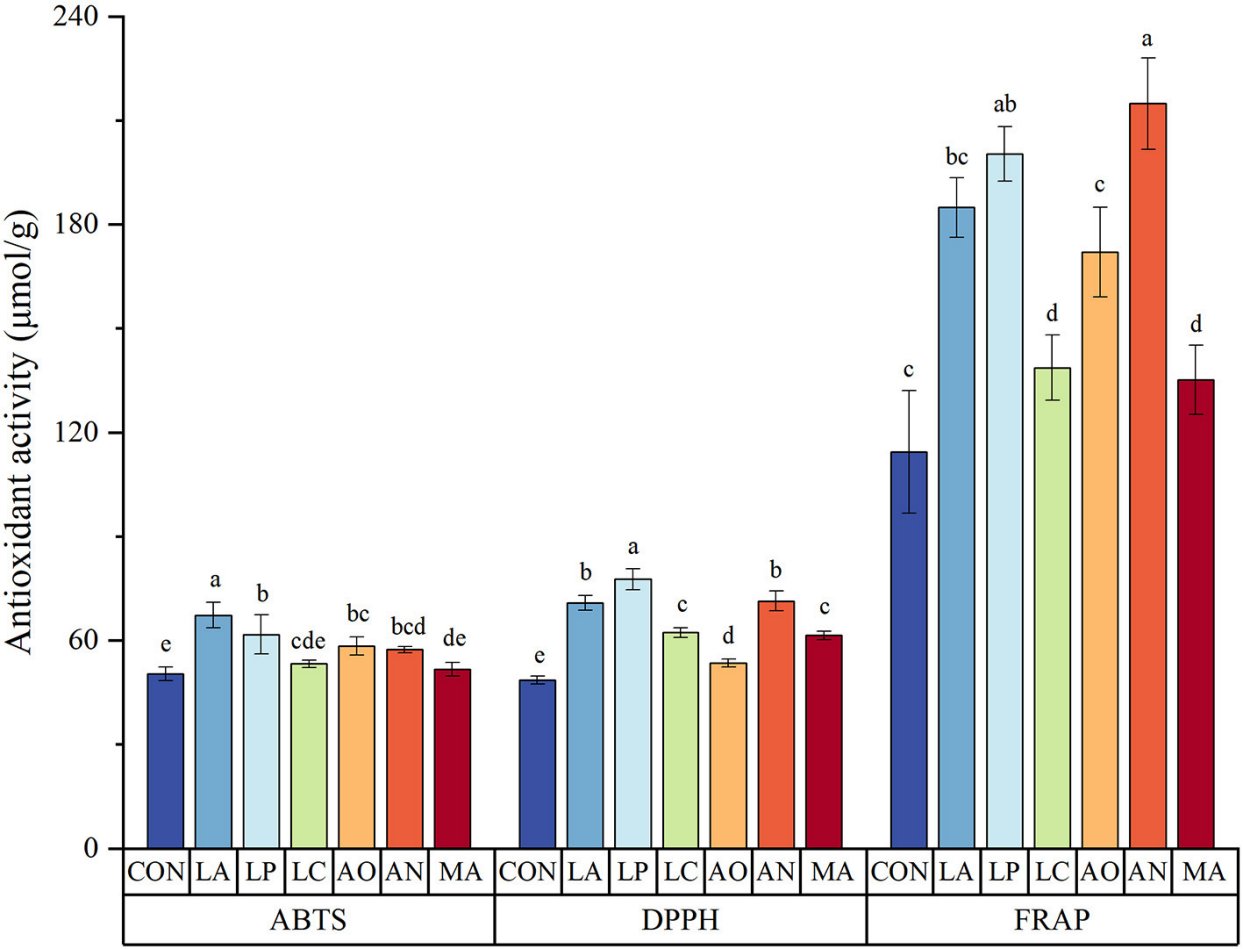
The antioxidant activity of blueberry pomace. Different letters in a column indicate significant differences (*p* < 0.05). Values are means of triplicate samples and expressed as mean ± SD. LA, *Lactobacillus acidophilus*; LP, *Lactobacillus plantarum*; LC, *Lactobacillus casei*; AO, *Aspergillus oryzae*; AN, *Aspergillus niger*; MA, *Monascus anka*.

The antioxidant activity of each sample was measured by ABTS, DPPH, and FRAP assays. LP exhibited the highest DPPH radical scavenging activity, reaching 77.66 μmol/g (Figure 1). The second highest DPPH radical scavenging activity was found in AN (71.37 μmol/g) and LA (70.85 μmol/g), with no significant difference between them. In comparison to the control group, the DPPH radical scavenging activities in the LP, AN, and LA groups were increased by 59.89, 46.94, and 45.87%, respectively.

In addition, SSF improved the FRAP radical scavenging activity of blueberry pomace. Similar to the DPPH results, the FRAP radical scavenging activities of all fermented blueberry pomace samples were also increased significantly compared with control by 18.20–87.82%. AN presented the highest FRAP radical scavenging activity, followed by LP. There was no significant difference between the FRAP radical scavenging activity of AN (214.79 μmol/g) or LP (200.33 μmol/g).

The results also demonstrated that the ABTS radical scavenging activity of all blueberry pomace samples was improved by SSF. LA (67.29 μmol/g) achieved the strongest ABTS radical scavenging activity, with an increase of 33.56%. Subsequently, the ABTS radical scavenging activities in the LP, AO, and AN groups were raised by 22.54, 15.93, and 13.86%, with no significant difference.

In general, *Lactobacillus plantarum, Lactobacillus acidophilus*, and *Aspergillus niger* showed greater antioxidant activities than other strains.

### Correlation analysis between polyphenol compounds and antioxidant activity

**Figure 2:**
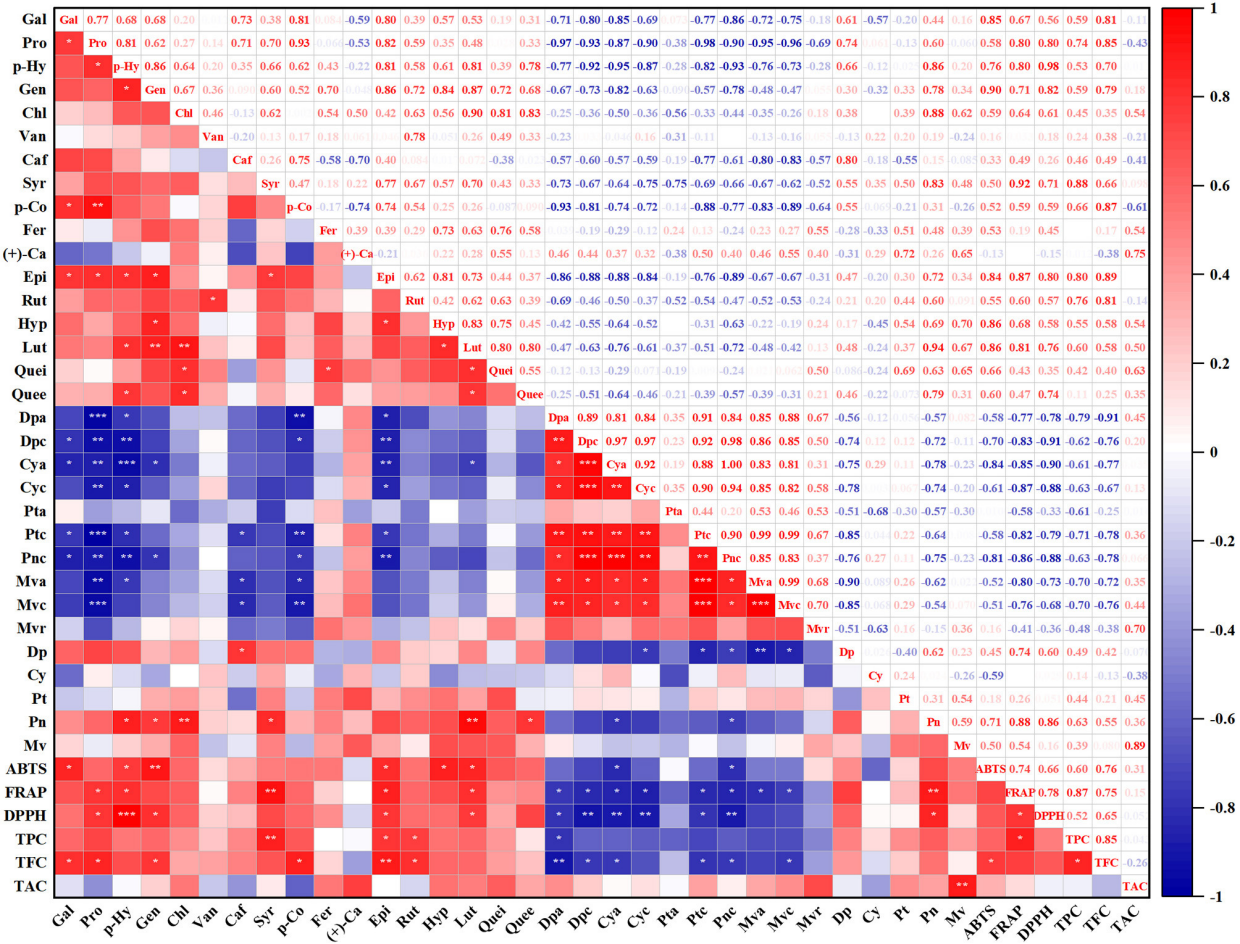
Pearson's correlation analysis of antioxidant activities and phytochemical content of blueberry pomace. *Denotes significant differences at *P* < 0.05, **denotes significant differences at *P* < 0.01, and ***denotes significant differences at *P* < 0.001. Gal, Gallic acid; Pro, Protocatechuic acid; *p*-Hy, *p*-Hydroxybenzoic acid; Gen, Gentisic acid; Chl, Chlorogenic acid; Van, Vanillic acid; Caf, Caffeic acid; Syr, Syringic acid; *p*-Co, *p*-Coumaric acid; Fer, Ferulic acid; (+)-Ca, (+)-Catechin Hydrate; Epi, Epicatechin; Rut, Rutin; Hyp, Hyperoside; Lut, Luteoloside; Quei, Quercitrin; Quee, Quercetin; Dpa, Delphinidin-3-O-galactoside; Dpc, Delphinidin-3-O-glucoside; Cya, Cyanidin-3-O-galactoside; Cyc, Cyanidin-3-O-glucoside; Pta, Petunidin-3-O-galactoside; Ptc, Petunidin-3-O–glucoside; Pnc, Peonidin-3-O-glucoside; Mva, Malvidin-3-O-galactosid; Mvc, Malvidin-3-O-glucoside; Mvr, Malvidin-3-O-arabinoside; Dp, Delphinidin; Cy, Cyanidin; Pt, Petunidin; Pn, Peonidin; Mv, Malvidin.

With the purpose of investigating the influence of fermentation on polyphenol compounds and antioxidant activity, Pearson's correlation analysis was conducted to assess the relationship between them. As shown in Figure 2, all anthocyanin substances were negatively correlated with gallic acid, protocatechuic acid, *p*-hydroxybenzoic acid, gentisic acid, caffeic acid, syringic acid, *p*-coumaric acid, epicatechin, and luteoloside, apart from petunidin-3-O-galactoside and malvidin-3-O-arabinoside. Similarly, TPC and TFC were negatively correlated with all anthocyanin substances as well. TAC was weakly negatively correlated with gallic acid, protocatechuic acid, vanillic acid, caffeic acid, *p*-coumaric acid, rutin, delphinidin, and cyanidin. In terms of anthocyanin and anthocyanidin, only delphinidin and peonidin were negatively correlated with all anthocyanin substances.

A significant positive correlation (*R*^2^ = 0.87) was found between TPC and FRAP. Furthermore, the contents of protocatechuic acid (*R*^2^ = 0.80), *p*-Hydroxybenzoic acid (*R*^2^ = 0.80), syringic acid (*R*^2^ = 0.92), gentisic acid (*R*^2^ = 0.71), epicatechin (*R*^2^ = 0.87), luteoloside (*R*^2^ = 0.81), delphinidin (*R*^2^ = 0.74), and peonidin (*R*^2^ = 0.88) were strongly positive correlated with FRAP radical scavenging activity as well. Additionally, the ABTS radical scavenging activity was considerably positively correlated with TFC (*R*^2^ = 0.76). Strongly positive correlations were also found between ABTS and gallic acid (*R*^2^ = 0.85), *p*-hydroxybenzoic acid (*R*^2^ = 0.76), gentisic acid (*R*^2^ = 0.90), epicatechin (*R*^2^ = 0.84), hyperoside (*R*^2^ = 0.86), and luteoloside (*R*^2^ = 0.81). The DPPH radical scavenging activity was significantly positively correlated with protocatechuic acid (*R*^2^ = 0.80), *p*-Hydroxybenzoic acid (*R*^2^ = 0.98), gentisic acid (*R*^2^ = 0.82), epicatechin (*R*^2^ = 0.80), luteoloside (*R*^2^ = 0.81), and peonidin (*R*^2^ = 0.86).

Thus, the findings of Pearson's correlation analysis revealed that the DPPH, ABTS, and FRAP radical scavenging activities were positively correlated with almost polyphenolic compounds, TFC, and TPC. Almost all anthocyanins were negatively correlated with the polyphenolic compounds.

### Principal component analysis

**Figure 3:**
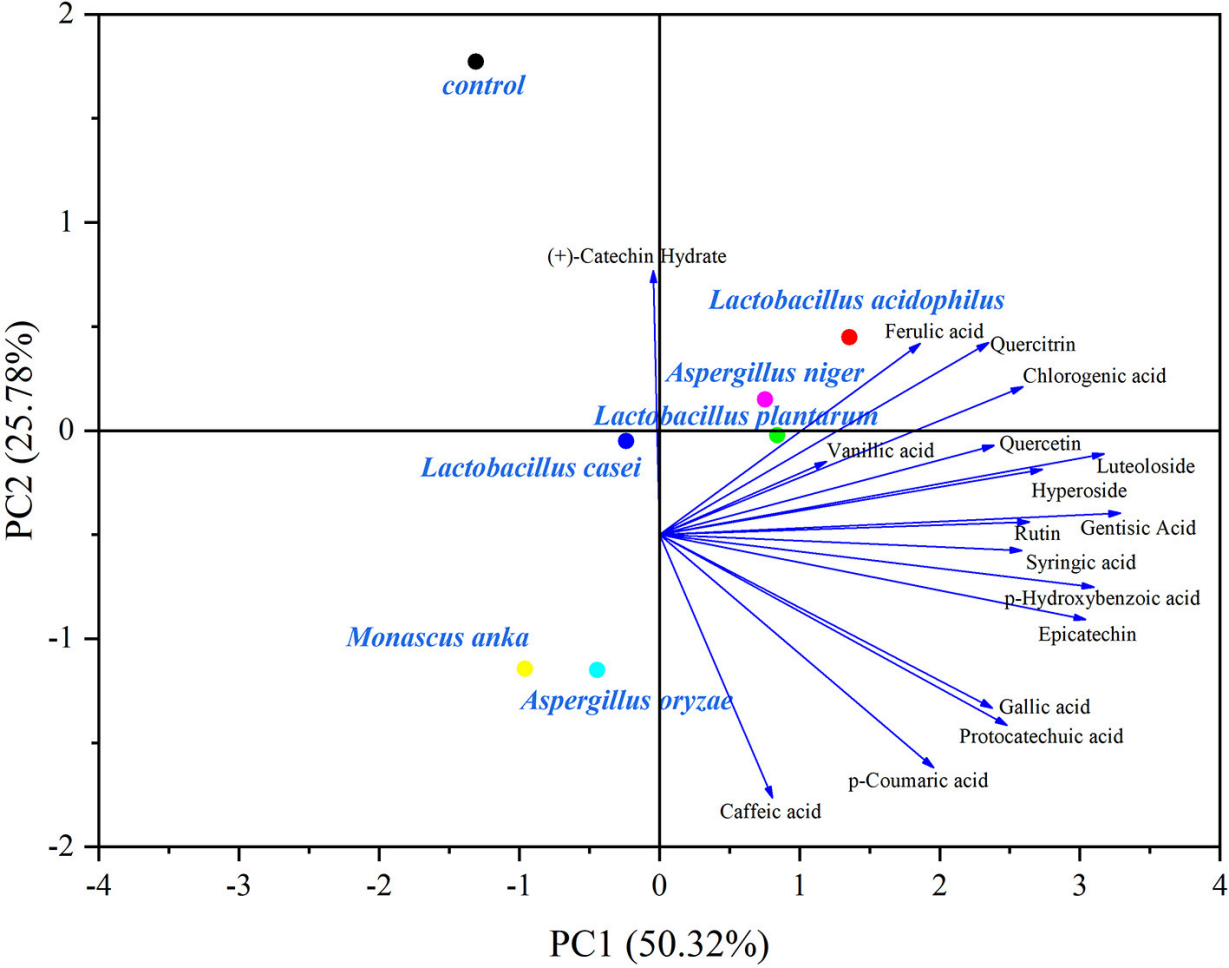
Biplot of the PCA of phenolic acid and flavonoid profiles in the blueberry pomace. Gal, Gallic acid; Pro, Protocatechuic acid; *p*-Hy, *p*-Hydroxybenzoic acid; Gen, Gentisic acid; Chl, Chlorogenic acid; Van, Vanillic acid; Caf, Caffeic acid; Syr, Syringic acid; *p*-Co, *p*-Coumaric acid; Fer, Ferulic acid; (+)-Ca, (+)-Catechin Hydrate; Epi, Epicatechin; Rut, Rutin; Hyp, Hyperoside; Lut, Luteoloside; Quei, Quercitrin; Quee, Quercetin.

**Figure 4:**
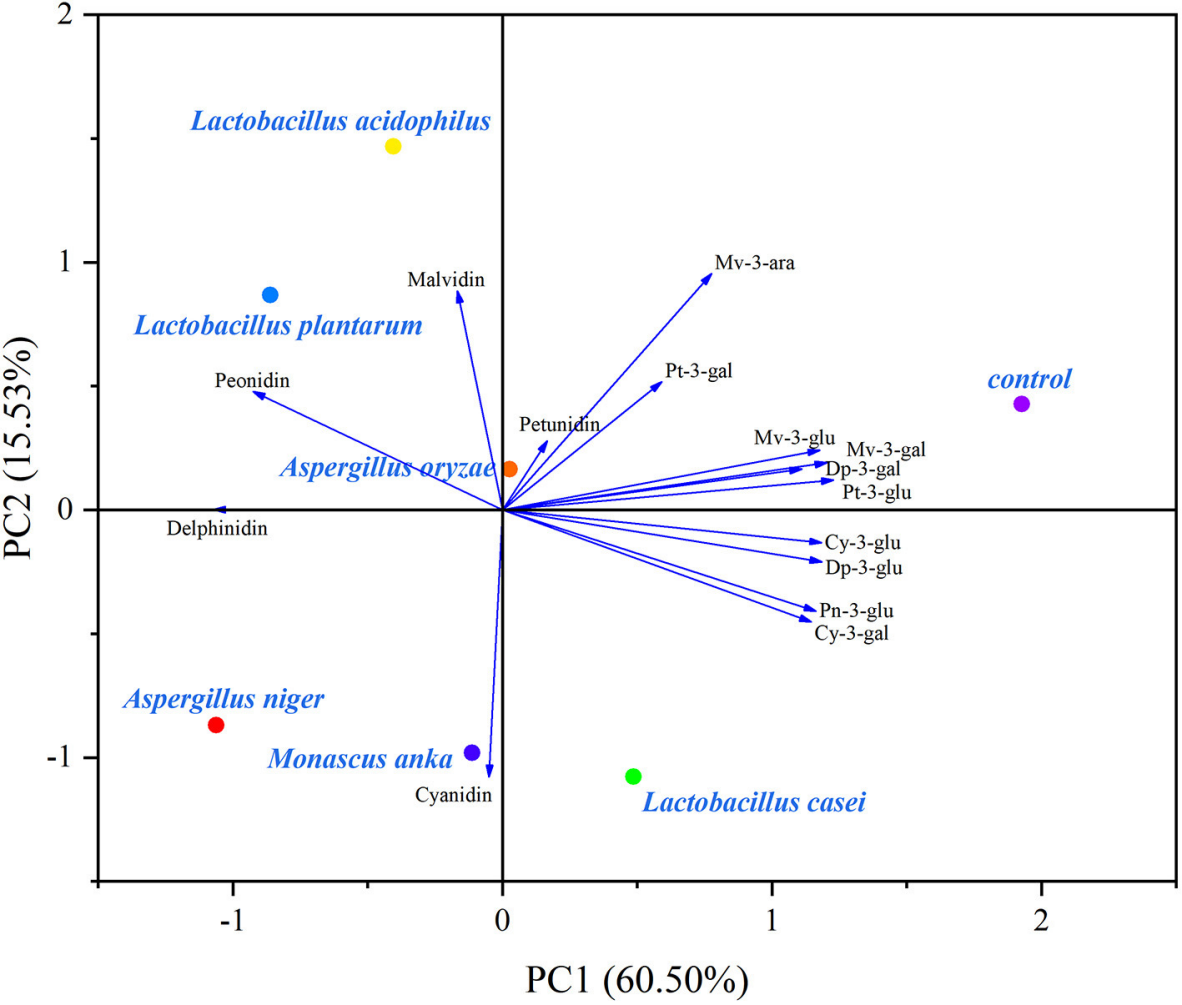
Biplot of the PCA of anthocyanin and anthocyanidin profiles in the blueberry pomace. Dp-3-gal, Delphinidin-3-O-galactoside; Dp-3-glu, Delphinidin-3-O-glucoside; Cy-3-gal, Cyanidin-3-O-galactoside; Cy-3-glu, Cyanidin-3-O-glucoside; Pt-3-gal, Petunidin-3-O-galactoside; Pt-3-glu, Petunidin-3-O–glucoside; Pn-3-glu, Peonidin-3-O-glucoside; Mv-3-gal, Malvidin-3-O-galactosid; Mv-3-glu, Malvidin-3-O-glucoside; Mv-3-ara, Malvidin-3-O-arabinoside.

PCA biplots exhibited changes in polyphenolic composition affected by SSF with different microbial strains (Figures 3, 4).

For the phenolic acid and flavonoid composition (Figure 3), the two principal components described 76.10% of the total variance in the evaluated data (50.32% for PC1 and 25.78% for PC2). The control group was located far away from all fermented samples, indicating that SSF significantly affected phenolic acids and flavonoids. All phenolic acids and flavonoids are distributed on the positive side of PC1, except for (+)-catechin. Similarly, LA, LP, and AN were distributed on the positive side of PC1, revealing that these strains had a positive influence on phenolic acid and flavonoid compounds. Additionally, LP and AN were close to each other, implying that the two strains had similar capacities for metabolizing those compounds.

In the case of anthocyanin composition (Figure 4), the two principal components described 76.03% of the total variance in the evaluated data (60.50% for PC1 and 15.53% for PC2). The control group was distant from fermented blueberry pomace, suggesting that anthocyanins and anthocyanidins were distinctly changed by SSF. All anthocyanins were located on the positive side of PC1, most anthocyanidins were distributed on the positive side of PC2. Additionally, the control group was close to anthocyanins and located on the positive side of PC1. On the contrary, most of fermented blueberry pomaces were situated on the negative side of PC1, implying that anthocyanins experienced a decreasing trend after SSF. This is shown in Table 1. According to Figure 4, the fermentation by LA, LP, and AN led to an increase in delphinidin, peonidin, and malvidin.

Overall, as shown in Figures 3, 4, *Lactobacillus acidophilus, Lactobacillus plantarum*, and *Aspergillus niger* had positive effects on polyphenolic compounds.

### Effect of SSF on bioaccessibility of polyphenol compounds in blueberry pomace

Bioaccessibility data of polyphenol compounds in blueberry pomace are presented in Table 4. The bioaccessibility of total phenolic acid, total flavonoid, and total anthocyanin in blueberry pomace was all enhanced with SSF. The bioaccessibility of total phenolic acid ranged from 55.34 to 67.75%. AN and LA had the highest bioaccessibility levels, with no significant difference, with increases of 22.43 and 18.47%, respectively. Similarly, Gao et al. (23) reported that the total phenol bioaccessibility of blueberry juice fermented by *Lactococcus lactis* and elevated by 34.23%. The bioaccessibility of total flavonoids varied from 13.32 to 20.91%. The highest total flavonoid bioaccessibility was observed in LA, increased by 56.98%. In the study by Morais et al. (24), the LA fermentation enhanced the bioaccessibility of flavonoid compounds in red pitaya pulp by 67.40–250%. The total anthocyanin bioaccessibility ranged from 1.59 to 2.71%, with AN exhibiting the largest increase, followed by LP and LA. There was no significant difference among those three groups. As a whole, lactic acid bacteria posed a higher effect on the bioaccessibility of polyphenolic compounds than fungi.

**Table 4:** Bioaccessibility of polyphenolic compounds in blueberry pomace (%).

	**Phenolic acids**	**Flavonoids**	**Anthocyanins**
Control	55.34 ± 2.73^d^	13.32 ± 0.52^f^	1.59 ± 0.16^c^
LA	65.56 ± 2.14^a^	20.91 ± 0.69^a^	2.49 ± 0.17^ab^
LP	61.42 ± 1.88^b^	16.89 ± 0.80^c^	2.63 ± 0.22^a^
LC	59.66 ± 1.79^bc^	16.47 ± 0.50^cd^	1.72 ± 0.18^c^
AO	57.03 ± 1.51^cd^	15.08 ± 0.41^de^	2.18 ± 0.23^b^
AN	67.75 ± 3.05^a^	19.56 ± 0.87^b^	2.71 ± 0.27^a^
MA	56.94 ± 1.16^cd^	14.21 ± 1.03^ef^	1.66 ± 0.25^c^

Different letters in a column indicate significant differences (*p* < 0.05). Values are means of triplicate samples and expressed as mean ± SD.

LA, *Lactobacillus acidophilus*; LP, *Lactobacillus plantarum*; LC, *Lactobacillus casei*; AO, *Aspergillus oryzae*; AN, *Aspergillus niger*; MA, *Monascus anka*

## Discussion

### The transformation of polyphenol compounds promoted by SSF

Anthocyanins are usually composed of glycosides and anthocyanidin by an α-linkage or β-linkage at C-3 of the anthocyanidin. Anthocyanidins were known as aglycones. The conversion of anthocyanins into small-molecule substances may be a general pattern. Anthocyanins initially could be degraded into anthocyanidins through the breakdown of glucoside bonds by glucosidase produced by microorganisms during fermentation (4, 25). Then, anthocyanidins appear for a very short time and are further metabolized to phenolic acids corresponding to anthocyanidin ring-B (26, 27). In our study, the significant decrease in anthocyanins was accompanied by the growth of anthocyanidins and phenolic acids, which was in general agreement with previous studies and validated the conversion of anthocyanins (23, 27, 28). Similarly, the increase in TPC and TFC and the drop in TAC further confirmed that SSF promoted the transformation of anthocyanins. These results were similar to those of earlier studies, in which the TPC and TFC of blueberry pomace and red bayberry pomace were increased after fermentation while TAC decreased (3, 15). Earlier studies showed that peonidin-3-glucoside had the greatest degradation rates compared with other anthocyanins (29). In our study, delphinidin-3-O-galactoside was degraded fastest, decreased by 41.52–65.39% compared with the control group. A drop in the content of glycosylated anthocyanins can lead to elevating their aglycone forms (anthocyanidins) (30). Correspondingly, delphinidin aglycon showed the greatest considerable extent of increase compared with other anthocyanidins in all fermented blueberry pomace samples. Delphinidin and peonidin were negatively correlated with all anthocyanins (Figure 2). However, cyanidin, petunidin, and malvidin were weakly associated with anthocyanins (Figure 2). This may be due to the instability of these aglycone forms, which are further degraded into phenolic acids and flavonoids.

The content of anthocyanin substances declined after fermentation, while the majority of phenolic acids and flavonoids presented a rise in the content (Tables 1, 2). The breakdown of anthocyanins could result in the accumulation of phenolic acids (28), which is supported by Pearson's correlation analysis (Figure 2). As shown in Figure 2, delphinidin-3-O-glucoside was strongly and negatively correlated with gallic acid (*R*^2^ = 0.87). This was consistent with the previous study reported by Ávila et al. (26), which revealed that the chemical breakdown of delphinidin-3-O-glucoside could lead to the generation of gallic acid. Furthermore, apart from petunidin-3-O-galactoside and malvidin-3-O-arabinoside, all anthocyanin substances were negatively correlated with gallic acid, protocatechuic acid, *p*-hydroxybenzoic acid, gentisic acid, caffeic acid, syringic acid, *p*-coumaric acid, epicatechin, and luteoloside (Figure 2). The results indicated that the increase in phenolic acid and flavonoid substances may be associated with the degradation of anthocyanins during fermentation, confirming that SSF promoted the transformation of polyphenols.

Compared with the control group, the contents of gallic acid, protocatechuic acid, *p*-hydroxybenzoic acid, gentisic acid, caffeic acid, *p*-coumaric acid, rutin, and epicatechin of six fermented blueberry pomaces were increased. While the content of (+)-catechin in all fermented groups was declined. This phenomenon may be attributed to the consumption of flavonoids by microbial strains (31). The difference in the content of these polyphenolic compounds among fermented blueberry pomaces may be due to the particular adaptability and ability of microbial strains to create carbohydrate-degrading enzymes (32). Only in the AN group, all anthocyanidins present a growth trend, with the highest concentration of all anthocyanidins also observed in the AN group. These results demonstrated that *Aspergillus niger* exerted a stronger capability for transforming anthocyanins to anthocyanidins than other strains. The highest TPC was also found in AN, indicating that the ability of *Aspergillus niger* to depolymerize intricate phenolic compounds into free forms was superior to that of other strains (32, 33).

### The antioxidant activity improved by SSF

The DPPH, ABTS, and FRAP radical scavenging activities of all fermented blueberry pomace samples were enhanced significantly compared with the control, suggesting that SSF considerably positively affected the antioxidant activity.

Numerous research studies have demonstrated that the rise in DPPH radical scavenging activity indicates that fermentation may improve the availability of some polyphenolic compounds with proton-donating properties (32, 34, 35). This is supported by the significantly strong positive correlations between the contents of protocatechuic acid (*R*^2^ = 0.80), *p*-Hydroxybenzoic acid (*R*^2^ = 0.98), gentisic acid (*R*^2^ = 0.82), and DPPH radical scavenging activity (Figure 2). According to earlier research studies, the elevation of DPPH radical scavenging activity might be due to the SSF process in grape pomace (36), apricot pomace (37), and chokeberry pomace (19).

Strong positive correlations were also found between ABTS and gallic acid (*R*^2^ = 0.85), *p*-hydroxybenzoic acid (*R*^2^ = 0.76), gentisic acid (*R*^2^ = 0.90), epicatechin (*R*^2^ = 0.84), hyperoside (*R*^2^ = 0.86), and luteoloside (*R*^2^ = 0.81) (Figure 2), which might be due to the conversion of ABTS•+ to ABTS by the phenolic hydroxyl groups (38). *p*-Hydroxybenzoic, epicatechin, and luteoloside exhibited a significantly strong positive correlation with DPPH, FRAP, and ABTS antioxidant activities, illustrating their vital contribution to the antioxidant potential of the fermented blueberry pomace.

The simpler structure of substances may contribute to their higher antioxidant activities (39). In our study, a strong negative correlation was generally observed between anthocyanins and antioxidant activities (Figure 2). Furthermore, the content of total anthocyanins was lower in the LA, LP, and AN groups than in the control groups, while the contents of total anthocyanidins and TPC were higher in the LA, LP, and AN groups. Hence, in the three antioxidant activity assays, blueberry pomace fermented by *Lactobacillus acidophilus, Lactobacillus plantarum*, and *Aspergillus niger* showed better antioxidant activity than other species.

### The bioaccessibility of polyphenol compounds enhanced by SSF

Polyphenolic compounds are generally recognized as having low bioaccessibility because of their insolubility, instability, and large molecular weight (38). Food matrix, food processing, and the interactions between polyphenols and other large molecular compounds may affect the absorption of polyphenols and influence their bioaccessibility (40). A growing number of reports have shown that microorganism fermentation positively affects the bioaccessibility of polyphenols (23, 24, 38, 41). Our results were in agreement with these studies. The bioaccessibility of total phenolic acids, total flavonoids, and total anthocyanins in blueberry pomace was enhanced by SSF. The bioaccessibility of the lactic acid bacteria groups was ranked at the forefront, while the bioaccessibility of the fungi groups was generally located in the posterior position except AN. This may be due to the different abilities of these microbial strains to metabolize and depolymerize polyphenols. Additionally, the bioaccessibility of total phenolic acid was remarkably higher than that of total flavonoid and total anthocyanin which may be related to their molecular size and cellular structure. Phenolic acids have smaller molecular sizes and simpler cellular structures, making them easier to absorb and metabolize compared to other polyphenolic compounds. As shown in Table 4, AN and LA always ranked in the top two places in all bioaccessibility assays, demonstrating that *Aspergillus niger* and *Lactobacillus acidophilus* enabled blueberry pomace with the higher bioaccessibility than other microbial strains.

## Conclusion

The present study demonstrated that SSF had notable effects on polyphenol profiles, antioxidant activity, and bioaccessibility of blueberry pomace. All of the TPC, TFC, and most of the individual phenolic acid substances and flavonoid substances in fermented blueberry pomaces were increased with SSF, while the individual anthocyanin substances and TAC showed a decreased tendency after SSF. An increase in all five individual anthocyanidin substances was only found in the AN group, which also showed the highest concentration of these substances. The stronger ability of strains to biotransform polyphenolic compounds contributed to a higher enrichment of antioxidant activity and bioaccessibility. Compared with other strains, *Lactobacillus acidophilus, Lactobacillus plantarum*, and *Aspergillus niger* showed better capacities for metabolizing polyphenolic compounds, leading to a greater increase in antioxidant activity and bioaccessibilityin fermented blueberry pomace. Therefore, the use of appropriate strains in SSF could enhance polyphenolic compounds and their bioaccessibilityin blueberry pomace.

## Data availability statement

The original contributions presented in the study are included in the article/supplementary material, further inquiries can be directed to the corresponding author.

## Author contributions

Z-XT: Conceptualization, Writing—original draft. Y-FL: Conceptualization, Writing—review and editing. M-XL: Writing—review and editing. QL: Writing—review and editing. XC: Visualization, Writing—review and editing. D-MH: Visualization, Writing—review and editing. Y-QR: Formal analysis, Software, Writing—review and editing.
